# Draft Genome Sequence of *Phyllobacterium endophythicum* mTS5, Isolated from *Lupinus micranthus* in Tunisia

**DOI:** 10.1128/MRA.00968-19

**Published:** 2019-11-07

**Authors:** Zoé Waller, Mokhtar Rejili, Mohamed Mars, Andreas Brachmann, Macarena Marín

**Affiliations:** a Genetics, Faculty of Biology, Ludwig-Maximilians-University Munich, Munich, Germany; b Laboratory of Biodiversity and Valorization of Arid Areas Bioresources (BVBAA), Faculty of Sciences, Gabès University, Gabès, Tunisia; University of Arizona

## Abstract

We report here the draft genome sequence of Phyllobacterium endophyticum mTS5, isolated from a *Lupinus micranthus* root nodule. The genome consists of 5,454,168 bp, with a GC content of 57%, and contains 5,676 protein-coding sequences.

## ANNOUNCEMENT

Lupines are emerging crops for human and livestock diets because of their protein- and fiber-rich seeds and their low requirements for nitrogen and phosphorus fertilization (1). Their ability to grow in nitrogen-poor soils is grounded in their symbiotic association with nitrogen-fixing bacteria (2). Thus, assessing the biodiversity of bacteria associated with lupines is of paramount importance for the use of these crops in sustainable agriculture.

Phyllobacterium endophyticum mTS5 was isolated from a single nodule on a Lupinus micranthus plant growing in the region of Takelsa, Tunisia. A whole nodule was dissected from a root and surface sterilized for 1 min with 95% ethanol and for 3 min with 4% sodium hypochlorite. The nodule was washed at least 5 times, and a 50-μl aliquot of the last washing suspension was checked for sterility on yeast mannitol agar (YMA) plates (3). The nodule was then separately crushed in a 20-μl aliquot of sterile water, and its content was streaked on YMA plates. These were incubated at 28°C under aerobic conditions for 5 to 7 days. Bacterial colonies were purified by repeated isolation at least 5 times.

Genomic DNA (gDNA) was extracted using the cetyltrimethylammonium bromide (CTAB) method (4) from a single-colony culture grown for 3 days in yeast mannitol broth. DNA was sheared on a Covaris M220 ultrasonicator with Covaris MicroCaps (50 μl) to approximately 600 bp. Two libraries were prepared using the sparQ DNA library prep kit (QuantaBio), according to the manufacturer’s instructions, with 600 ng and 107 ng of sheared gDNA, respectively. Libraries were quality controlled with a high-sensitivity DNA kit on a Bioanalyzer instrument (Agilent) for amplicon sizes between 300 and 800 bp and absence of adapter primers and then quantified on a Qubit 2.0 fluorometer (with a double-stranded DNA [dsDNA] high-sensitivity [HS] assay kit; Thermo Fisher Scientific). The genome was sequenced in the Genomics Service Unit (LMU Biocenter, Munich, Germany) on an Illumina MiSeq system with v3 chemistry (five partial rounds of paired-end sequencing with 2 × 250 bp or 2 × 300 bp), generating 5,765,152 paired-end reads. Adapter and quality trimming (trim limit, 0.01) resulted in 2,810,080 paired-end reads with an average length of 183 bp, from which the genome was assembled *de novo* using CLC Genomics Workbench 9.0 (Qiagen). Default parameters were used for all software unless otherwise specified. Quality control of the assembly was performed using QUAST 5.0.2 (5). In total, 93 contigs were obtained, with 5,454,168 bp of sequence information and 93-fold coverage. The average contig length was 58,081 bp, with the largest contig being 366,285 bp and the shortest being 511 bp. The *N*_50_ value was 165,103 bp, and the GC content 57%. The public version of the genome was annotated with the NCBI Prokaryotic Genome Annotation Pipeline (PGAP) (6), while the functional annotation of the in-house version was performed using the Rapid Annotations using Subsystems Technology (RAST) server for homology search against the SEED database (7). For the latter, in total, 5,676 protein-coding sequences and 49 RNA genes were predicted. A BLASTn search using the *nodC* and *nifH* orthologs of Phyllobacterium sophorae CCBAU 03422 (GenBank accession numbers KJ685943 and KJ685942, respectively) and Mesorhizobium japonicum MAFF303099 (accession number BA000012) against the *Phyllobacetrium endophyticum* mTS5 genome revealed the lack of the *nod* and *nif* symbiotic clusters in this strain.

A pairwise comparison based on the 16S rRNA and *recA* gene sequences was generated in CLC Main Workbench 7.0 (Qiagen). The type strain Phyllobacterium endophyticum PEPV15 was identified as the closest strain. Average nucleotide identity analysis was performed using the OrthoANIu algorithm (8). The obtained value, 98.56%, is above the species cutoff (9), which supports systematic placement of strain mTS5 in the species Phyllobacterium endophyticum.

### Data availability.

The draft genome sequence has been deposited at NCBI/GenBank under BioProject number PRJNA559855, BioSample number SAMN12561461, accession number VOWA00000000, and SRA numbers SRR9985975 and SRR9985976.
